# Calcium Modulation, Anti-Oxidant and Anti-Inflammatory Effect of Skin Allergens Targeting the Nrf2 Signaling Pathway in Alzheimer’s Disease Cellular Models

**DOI:** 10.3390/ijms21207791

**Published:** 2020-10-21

**Authors:** Ana Silva, Marta Pereira, Mylène A. Carrascal, Gonçalo Brites, Bruno Neves, Patrícia Moreira, Rosa Resende, Maria Manuel Silva, Armanda E. Santos, Cláudia Pereira, Maria Teresa Cruz

**Affiliations:** 1 Center for Neuroscience and Cell Biology and Institute for Biomedical Imaging and Life Sciences, University of Coimbra, 3000-548 Coimbra, Portugal; patriciaraquel_jm@hotmail.com (P.M.); rositaresende@gmail.com (R.R.); msilva@ff.uc.pt (M.M.S.); aesantos@ci.uc.pt (A.E.S.); cpereira@cnc.cj.uc.pt (C.P.); 2 Faculty of Medicine, University of Coimbra, 3000-548 Coimbra, Portugal; martarosendopereira@gmail.com; 3 Tecnimede Group, 2710-089 Sintra, Portugal; mylenecarrascal87@gmail.com; 4 Faculty of Pharmacy, University of Coimbra, 3000-548 Coimbra, Portugal; g.sousabrites3@gmail.com; 5 Department of Medical Sciences and Institute for Biomedicine (iBiMED), University of Aveiro, 3810-193 Aveiro, Portugal; bruno.neves@ua.pt; 6 University of Coimbra, Institute for Interdisciplinary Research (IIIUC), 3030-789 Coimbra, Portugal

**Keywords:** skin allergens, Nrf2, redox status, calcium, mitochondria, neuroinflammation

## Abstract

Experimental evidence highlights nuclear factor (erythroid-derived 2)-like 2 (Nrf2) as a molecular target in Alzheimer’s disease (AD). The well-known effect of electrophilic cysteine-reactive skin allergens on Nrf2-activation led to the hypothesis that these compounds could have a therapeutic role in AD. This was further supported by the neuroprotective activity of the skin allergen dimethyl fumarate (DMF), demonstrated in in vivo models of neurodegenerative diseases. We evaluated the effect of the cysteine-reactive allergens 1,4-phenylenediamine (PPD) and methyl heptine carbonate (MHC) on (1) neuronal redox imbalance and calcium dyshomeostasis using N2a wild-type (N2a-wt) and human APP-overexpressing neuronal cells (wild-type, N2a-APPwt) and (2) on neuroinflammation, using microglia BV-2 cells exposed to LPS (lipopolysaccharide). Phthalic anhydride (PA, mainly lysine-reactive), was used as a negative control. DMF, PPD and MHC increased Hmox1 gene and HMOX1 protein levels in N2a-APPwt cells suggesting Nrf2-dependent antioxidant activity. MHC, but also PA, rescued N2a-APPwt mitochondrial membrane potential and calcium levels in a Nrf2-independent pathway. All the chemicals showed anti-inflammatory activity by decreasing iNOS protein in microglia. This work highlights the potential neuroprotective and anti-inflammatory role of the selected skin allergens in in vitro models of AD, and supports further studies envisaging the validation of the results using in vivo AD models.

## 1. Introduction

Currently, AD is the most common form of dementia accounting for about 60–70% of global neurodegenerative diseases and is the fifth leading cause of death worldwide among the elderly population [1,2]. A disease-modifying treatment is of utmost importance for AD not only for proper care and management of the affected patients, but also to reduce society’s socioeconomic burden. As reviewed by Dong et al. (2019) there are no therapeutic drugs available to date to prevent or treat the pathology, thus AD is still a progressive and fatal disease [3]. The comprehension of the molecular basis of disease, as well as the possible mechanisms of therapy failure, were recognized as crucial for the identification of new targets and further development of innovative therapeutic strategies. Indeed, some AD pathological hallmarks, like neuroinflammation and impaired redox balance, could be therapeutic targets for disease management and recent reports have highlighted the protective role of Nrf2 activation in reducing oxidative stress in both in vitro and in vivo models of neurodegenerative diseases such as AD [4,5,6]. Accordingly, it has been reported that in AD, the Nrf2 localization in hippocampal neurons is mainly cytoplasmatic, suggesting an impaired translocation to the nucleus and consequent Nrf2 pathway dysfunction [4]. Furthermore, we recently demonstrated that oxidative stress involving changes in Nrf2 is present in early stages of AD, which leads to repression of antioxidant defense expression, perpetuating the disease [7]. This leads to a neuroinflammatory status, not only due to microglia activation but also to decreased Nrf2 activity. Additionally, the improvement of several characteristic symptoms of this disease including cognitive function was seen in AD mice models after Nrf2 modulation [6]. Altogether, these studies provide a compelling rationale for targeting Nrf2 as a therapeutic strategy to reinforce endogenous brain defense mechanisms against AD-associated pathological hallmarks.

Skin allergens possessing electrophilic properties have been shown to activate Nrf2, which is well explained by the direct reactivity of most sensitizing chemicals to key cysteine residues of kelch-like erythroid cell-derived protein with cap‘n’collar homology-associated protein 1 (Keap1) [8], leading to its dissociation from Nrf2. After Keap1 dissociation, Nrf2 translocates to the nucleus to upregulate genes that encode proteins mostly involved in detoxification [9,10,11]. Of notice, DMF (dimethyl fumarate), a fumaric acid ester, is a new orally available disease-modifying agent that was recently approved by the FDA and EMA as a first-line therapy for the management of relapsing forms of multiple sclerosis [12,13], a disease that shares several pathophysiological characteristics with AD. DMF is a skin allergen known to react with cysteine residues [14] and was recently demonstrated to prevent spatial memory impairments and hippocampal neurodegeneration in rats [15]. Therefore, the well-established effect of skin allergens on Keap1-Nrf2-antioxidant response element (ARE) regulatory pathway activation, concomitantly with experimental evidence highlighting Nrf2 as an attractive molecular target in the context of AD, led us to hypothesize that skin allergens may have a positive and therapeutic role in AD. Although skin sensitizers may trigger an allergic response after skin interaction, these Nrf2-activating molecules could have beneficial effects after oral or intranasal administration, similarly to DMF [14,16,17]. Considering the above, the present study aims to investigate whether DMF and other unexplored electrophilic skin allergens could have a preventive and/or therapeutic role in AD. To tackle this goal, in a first approach, an extensive physical library of allergens was screened in terms of hydrophobicity, drug-likeness properties and toxicity prediction in order to select a set of compounds able to be absorbed at the gastrointestinal tract, to cross the BBB (blood–brain barrier) and to exert low to moderate toxicity. Besides DMF (as a positive control), we selected two drug-like electrophiles unexplored in the context of neurodegenerative diseases, PPD (1,4-phenylenediamine) and MHC (methyl heptine carbonate) that, similarly to DMF, react with cysteine residues. PA (phthalic anhydride), which mainly reacts with lysine residues, was used as a negative control. The antioxidant and anti-inflammatory properties of these molecules were studied in vitro, using: (1) a mouse neuronal cell line (N2a) either wild-type (N2a-wt) or overexpressing human wild-type APP (N2a-APPwt), as an AD neuronal model, and (2) a mouse microglia cell line (BV-2) stimulated with lipopolysaccharide (LPS), as an inflammation model. The results gathered in this work demonstrated that DMF, PPD and MHC increased Hmox1 gene expression and HMOX1 protein levels in N2a-APPwt cells suggesting Nrf2-dependent antioxidant activity. MHC, but also PA, diminished mitochondrial calcium levels and rescued mitochondrial membrane potential in N2a-APPwt cells demonstrating that mitochondrial protection by electrophiles is independent of its reactivity towards cysteine residues and, therefore, of Nrf2 activation. All the chemicals studied showed anti-inflammatory activity by decreasing iNOS protein expression in LPS-exposed microglia. Overall, this work highlights the potential neuroprotective and anti-inflammatory role of the selected skin allergens in in vitro models of AD, which would not normally be considered useful in drug discovery programs. Nevertheless, further studies envisaging the validation of the results using in vivo AD models are needed.

## 2. Results

### 2.1. Selection of Skin Allergens

The sensitizing potentials of skin allergens have been evaluated based on compiled data from the local lymph node assay (LLNA) EC3%, which measures a chemical’s capacity in stimulating cell proliferation of mouse lymph nodes. Accordingly, skin allergens are classified as extreme, strong, moderate, weak and very weak/non-sensitizers [18,19,20]. Basketter et al. (2014) have categorized skin allergens according to their human sensitizing potency, which separates compounds into six potency categories [21]. Focused on this knowledge, molecules of categories 1, 2 and 3 [21] and of extreme, strong and moderate [18,19,20], were collected (Supplementary Table S1). When available, information concerning the reactivity of skin allergens towards cysteine and lysine residues was also gathered, based on the peptide reactivity assay, according to the score ranging from 0 to 4 (Supplementary Table S2) as proposed by Natsch et al. (2009) [19]. This information is of utmost importance since skin allergens possessing electrophilic properties have been shown to activate Nrf2 through their direct reactivity towards cysteine residues of Keap1, leading to its dissociation from Nrf2. Then, Nrf2 translocates to the nucleus, inducing the transcription of several protective genes (reviewed by Natsch, 2010) [8].

In order to select a set of compounds that are able to be absorbed at the gastrointestinal tract, to cross the blood–brain barrier (BBB) and to exert low to moderate toxicity, the allergens were examined for hydrophobicity, drug-likeness properties and toxicity prediction (as described in Materials and Methods section). The chemicals that showed PAINS51 were excluded and the remaining chemicals were filtered to high oral absorption and the ability to cross the BBB (Table 1) according to the Swiss ADME online predictor.

Hence, four drug-like electrophilic compounds were selected, from categories 1 and 2 (human data) and classified as extreme or strong (LLNA data). DMF was used as a positive control since it is a Nrf2 activator and has been shown to exert beneficial effects in the context of neurodegenerative diseases [22,23]. For negative control we chose PA, a skin sensitizer with low cysteine reactivity. Two of these are unexplored electrophilic chemicals in the context of neurodegenerative diseases, PPD and MHC, which are both highly reactive towards cysteine residues.

### 2.2. Effect of Skin Allergens on N2a and BV-2 Cell Metabolism and Viability

**Figure 1:**
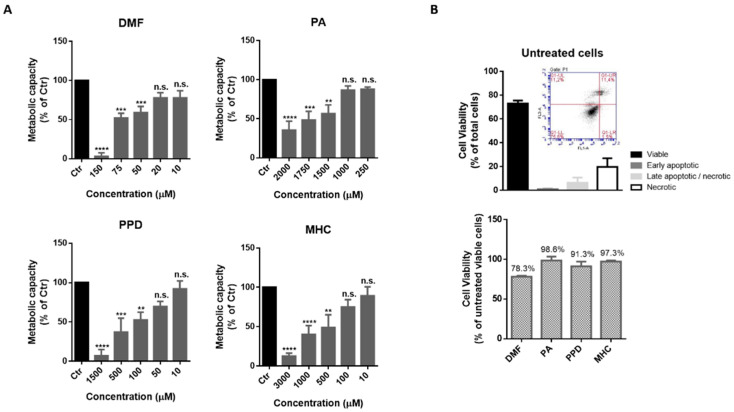
Determination of a non-toxic concentration of the chemicals in neuronal cells. (**A**) Metabolic capacity of N2a-wt cells exposed to skin allergens at different concentrations for 24 h and evaluated by the Alamar Blue assay. (**B**) N2a-wt viability after exposure to 14 µM of DMF, 500 µM of PA, 10 µM of PPD and 100 µM of MHC for 24 h. The cells were after stained for Annexin V (apoptotic cells marker) and 7-AAD (necrotic cells marker) and analyzed by flow cytometry. Similar results regarding the number of cells (% of total) of viable, apoptotic and necrotic cell populations were obtained for untreated (depicted in (**B**), upper graph), DMF, PA, PPD and MHC treated cells (Supplementary Figure S1). Bars in the lower graph in (**B**) show the percentage of viable cells considering viability of untreated cells as 100%. Values are the mean ± SEM of six (**A**) or three (**B**) independent experiments and expressed as the percentage of untreated (control, Ctr) cells. Statistics: One-way ANOVA with Dunnett’s multiple comparisons test. *p* < 0.05 was considered significant: ** *p* < 0.01, *** *p* < 0.001 and **** *p* < 0.0001, compared to Ctr. n.s.—non-significant. Legend: DMF—Dimethyl fumarate, PA—Phthalic anhydride, PPD—1,4-Phenylenediamine, MHC—Methyl heptine carbonate.

**Figure 2:**
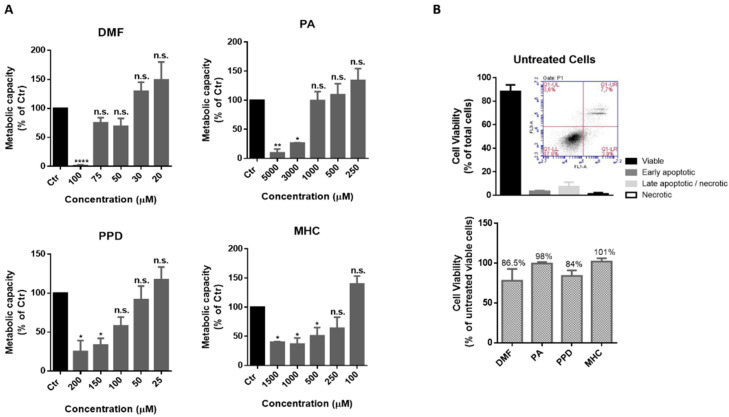
Determination of a non-toxic concentration of the chemicals in microglia cells. (**A**) Metabolic capacity of BV-2 cells exposed to skin allergens at different concentrations for 24 h and evaluated by the Alamar Blue assay. (**B**) BV-2 viability after exposure to 30 µM of DMF, 500 µM of PA, 25 µM of PPD and 200 µM of MHC for 24 h. The cells were after stained for Annexin V (apoptotic cells marker) and 7-AAD (necrotic cells marker) and analyzed by flow cytometry. Similar results regarding the number of cells (% of total) of viable, apoptotic and necrotic cell populations were obtained for untreated (depicted in (**B**), upper graph), DMF, PA, PPD and MHC treated cells (Supplementary Figure S2). Bars in the lower graph (**B**) show the percentage of viable cells considering viability of untreated cells as 100%. Values are the mean ± SEM of seven (**A**) or three (**B**) independent experiments and expressed as the percentage of untreated (control, Ctr) cells. Statistics: One-way ANOVA with Dunnett’s multiple comparisons test. *p* < 0.05 was considered significant: * *p* < 0.05, ** *p* < 0.01 and **** *p* < 0.0001, compared to Ctr. n.s.—non-significant. Legend: DMF—Dimethyl fumarate, PA—Phthalic anhydride, PPD—1,4-Phenylenediamine, MHC—Methyl heptine carbonate.

In order to find a non-toxic concentration of chemicals to be further tested in neuronal and microglia cells, N2a-wt (Figure 1) and BV-2 (Figure 2) cells were incubated with different concentrations of the selected skin sensitizers, for 24 h, and the cell’s metabolic capacity was evaluated through the Alamar blue assay (Figure 1A and Figure 2A). Accordingly, we selected concentrations of the tested chemicals that did not induce significant alterations in N2a-wt or BV-2 cell metabolic activity. Cell survival was further evaluated by flow cytometry using Annexin V (apoptotic cell marker) and 7-AAD (necrotic cell marker), in order to disclose viable, apoptotic and necrotic cell populations (Figure 1B and Figure 2B). Considering untreated cells as 100% viable (Figure 1B and Figure 2B, lower graph), we can conclude that the selected concentrations did not evoke a drastic percentage of cell death (up to ~20%) and, based on this rational, the following concentrations were selected for the subsequent studies in N2a cell lines: 14 µM DMF, 500 µM PA, 10 µM PPD and 100 µM MHC; and in BV-2 cell line: 30 µM DMF, 500 µM PA, 25 µM PPD and 200 µM MHC. These and subsequent results are compared to untreated control (Ctr) cells since the concentration of DMSO used (up to 0.05%), did not affect the assays regarding Nrf2 activation (Supplementary Figure S3) or any of the biological parameters evaluated in this study (data not shown).

### 2.3. Antioxidant Response Evoked by Skin Allergens in N2a Neuronal Cells

**Figure 3:**
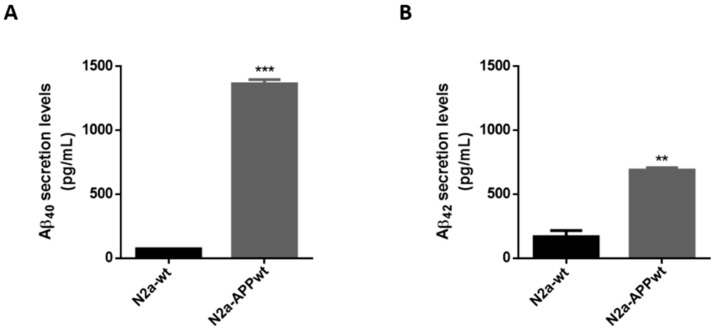
Aβ40 and Aβ42 peptide levels in N2a neuronal cells. Aβ40 (**A**) and Aβ42 (**B**) levels quantified in N2a serum-free supernatants, using a commercial kit. Values are the mean ± SEM of two independent experiments and expressed in pg/mL. Statistics: Unpaired *t*-test. *p* < 0.05 was considered significant: ** *p* < 0.01 and *** *p* < 0.001, compared to N2a-wt.

One of the features of AD is the generation of Aβ peptides. Hence, to confirm that APP-overexpressing cells did, in fact, secrete Aβ40 and Aβ42 peptides, we measured both species levels in N2a-wt and N2a-APPwt cells. As expected, N2a-APPwt cells secreted significantly higher levels of Aβ40 (~18 fold-increase; *p* < 0.0001) and Aβ42 (~4 fold-increase; *p* < 0.01), compared to N2a-wt (Figure 3), thus supporting its use as an AD cellular model. 

#### 2.3.1. Skin Allergens Activate Nrf2 Transcription Factor in APP-Overexpressing Neuronal Cells

**Figure 4:**
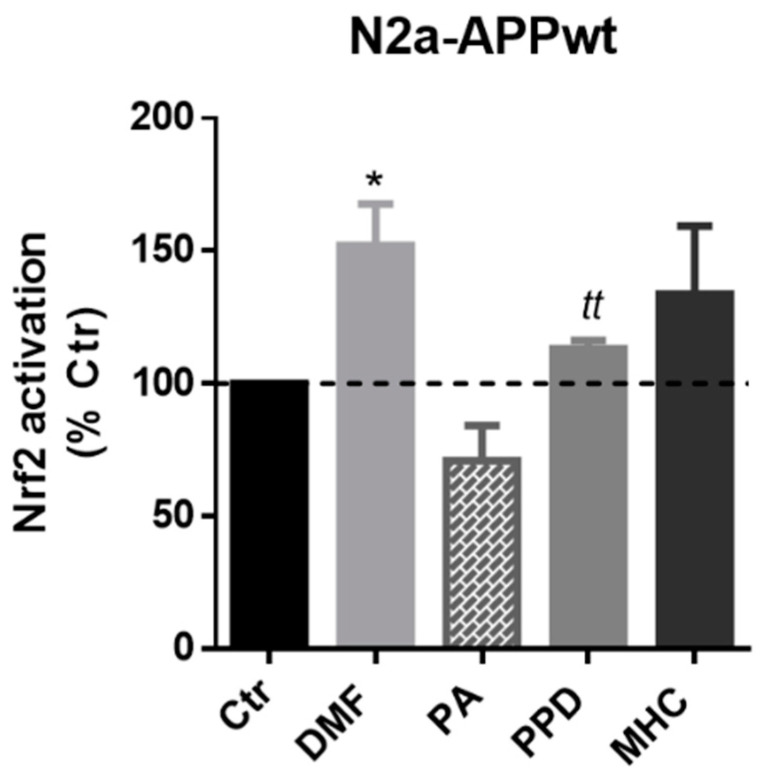
Effect of skin allergens on Nrf2 transcription factor activation in APP overexpressing-cells. Nrf2 activation determined in N2a-APPwt cells after chemical exposure for 1 h. Results are expressed as percentage of untreated control (Ctr) cells. Values are the mean ± SEM of three independent experiments. Statistics: One-way ANOVA with Dunnett’s multiple comparisons test (*) and unpaired *t*-test (*t*). *p* < 0.05 was considered significant: * p < 0.05 and *^tt^ p* < 0.01, compared to Ctr. Legend: DMF—Dimethyl fumarate, PA—Phthalic anhydride, PPD—1,4-Phenylenediamine, MHC—Methyl heptine carbonate.

Next, we wanted to determine if the selected skin allergens were able to activate Nrf2 in our AD cell model. Hence, we incubated N2a-APPwt cells with the previously determined concentrations of the chemicals for 1 h and 4 h. Preliminary results suggested a Nrf2 activation decrease in N2a-APPwt cells, after 4 h of PPD and MHC exposure (Supplementary Figure S3A), which was also observed in N2a-wt cells (Supplementary Figure S3C,D). Thus, we chose to evaluate Nrf2 activation in N2a-APPwt cells after 1 h of allergens exposure (Figure 4). According to our results, all the allergens tested induced Nrf2 activation in APP-overexpressing cells (except the negative control, PA), being statistically significant for both DMF and PPD (Figure 4). 

#### 2.3.2. Skin Allergens Increased Hmox1 Gene Expression in APP-Overexpressing Neuronal Cells

**Figure 5:**
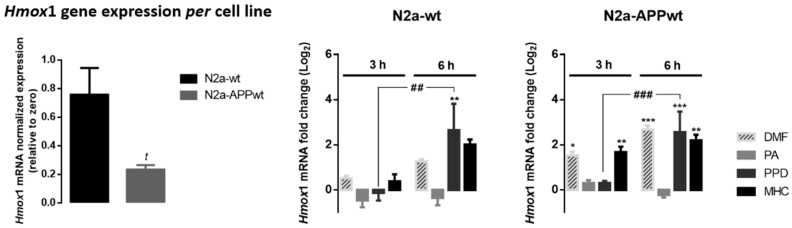
Effect of skin allergens on Hmox1 gene expression in neuronal cells. Normal (N2a-wt) and APP-overexpressing cells (N2a-APPwt) were exposed to the chemicals for 3 h or 6 h and mRNA levels of Hmox1 were analyzed by RT-PCR. Values are the mean ± SEM of three independent experiments and expressed relatively to control cells (log_2_ = 0). Statistics: One-way ANOVA with Tukey’s multiple comparisons test. *p* < 0.05 was considered significant: * p < 0.05, ** *p* < 0.01 and *** *p* < 0.001, compared to control cells; *^##^ p* < 0.01 and ^###^ *p* < 0.001, compared to 6 h of chemical exposure. Basal mRNA levels of Hmox1 in untreated N2a neuronal cell lines are depicted in the left graph. The bars correspond to the normalized Hmox1 expression, expressed relatively to zero. Values are the mean ± SEM of seven independent experiments. Statistics: Unpaired *t*-test. *^t^ p* < 0.05, compared to N2a-wt cells. Legend: DMF—Dimethyl fumarate, PA—Phthalic anhydride, PPD—1,4-Phenylenediamine, MHC—Methyl heptine carbonate.

As widely known, in stressed neural tissues, Hmox1 gene induction is highly dependent on Nrf2 activation and plays an important role in the antioxidant and anti-inflammatory cellular mechanisms [24,25]. Thus, to perceive how skin allergen exposure modulates the basal levels of Hmox1 gene in neuronal cells, the N2a-wt and N2a-APPwt cells were incubated in the absence or in the presence of skin allergens, for 3 and 6 h, and the mRNA levels were analyzed by real-time RT-PCR (Figure 5). 

According to the results obtained, N2a-wt cells express significantly higher levels of Hmox1 mRNA relatively to N2a-APPwt (Figure 5, left graph), which is in accordance with a Nrf2-deficient pathway observed in AD. As expected, DMF increased Hmox1 gene expression in N2a-APP-overexpressing cells, which was observed right after 3 h of cell treatment. Of note, similar results were obtained in cells exposed to MHC, while exposure to PPD induced a Hmox1 gene expression increase only after 6 h of treatment (Figure 5, N2a-APPwt). PA did not evoke alterations in Hmox1 gene expression at the time points tested, which might be related to the fact that this molecule is more prone to react with lysines than with cysteines, hence not efficiently activating the Nrf2 pathway.

#### 2.3.3. Skin Allergens Increased HMOX1 Protein Levels in APP-Overexpressing Neuronal Cells

**Figure 6:**
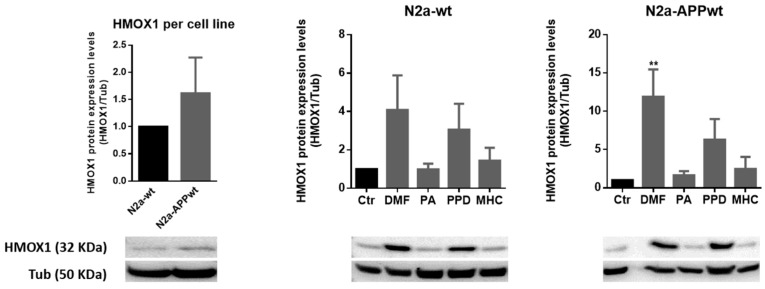
Effect of skin allergens on HMOX1 protein levels in neuronal cells. Western blot analysis of HMOX1 protein levels in N2a-wt and N2a-APP-overexpressing cells treated with the chemicals, for 24 h. Representative images of the blots are shown. Values are the mean ± SEM of three independent experiments normalized for tubulin (Tub) and expressed relatively to control (Ctr) cells. Statistics: One-way ANOVA with Dunnett’s multiple comparisons test. *p* < 0.05 was considered significant: ** *p* < 0.01 compared to Ctr. Basal levels of HMOX1 protein in untreated N2a neuronal cell lines are depicted in the left graph and expressed relatively to N2a-wt cells. Legend: DMF—Dimethyl fumarate, PA—Phthalic anhydride, PPD—1,4-Phenylenediamine, MHC—Methyl heptine carbonate.

Since gene transcription does not always linearly correlate with its target protein expression [26], we wanted to confirm if the chemical-induced increase in the Hmox1 gene (Figure 5) resulted in higher protein levels. Thus, we analyzed HMOX1 protein expression by Western blotting in N2a-wt and APP-overexpressing cells after 24 h of chemical exposure (Figure 6). 

As observed in Figure 6, all the molecules tested induced an increase in HMOX1 protein levels, with exception of PA, with this increase statistically significant for DMF in N2a-APPwt. Although not significant, both PPD and MHC induced a ~6-fold and ~2.5-fold increase of HMOX1 protein levels in N2a-APPwt cells. These results follow the same pattern of those obtained for Hmox1 gene expression (Figure 5), thus sustaining the beneficial effect of the molecules tested in activating Nrf2 antioxidant via a neuronal AD cell model. Representative immunocytochemistry images of equally treated cells stained against HMOX1 are shown in the Supplementary Materials (Figure S4). 

**Figure 7:**
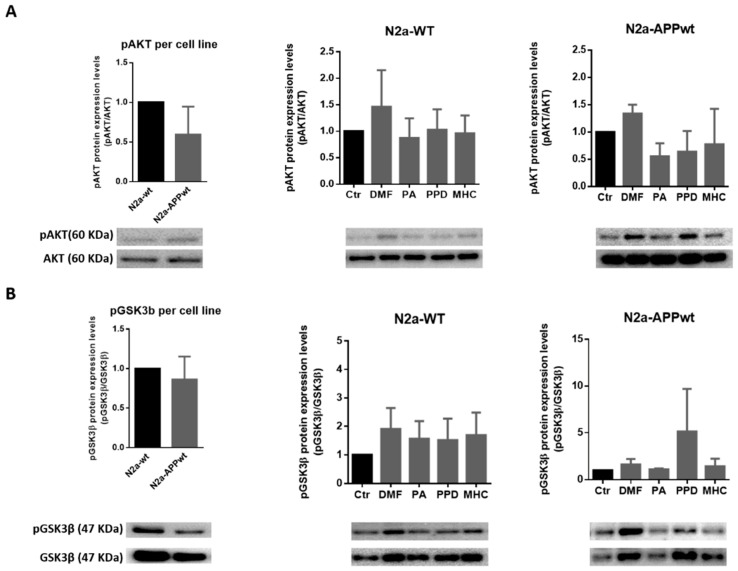
Effect of skin allergens on AKT activation and GSK3β inactivation in neuronal cells. Western blot analysis of AKT (**A**) and GSK3β (**B**) phosphorylated (p) protein levels in N2a-wt and N2a-APP-overexpressing cells treated with the chemicals for 24 h. Values are the mean ± SEM of three independent experiments and were expressed relatively to control (Ctr) cells. The basal levels of pAKT and pGSK3β in untreated N2a neuronal cell lines are depicted in the left graph and expressed relatively to N2a-wt cells. Legend: DMF—Dimethyl fumarate, PA—Phthalic anhydride, PPD—1,4-Phenylenediamine, MHC—Methyl heptine carbonate.

DMF was previously shown to exert a double mechanism of Nrf2 activation by disrupting the KEAP1/Nrf2 interaction and through the glycogen synthase kinase 3 beta (GSK3β)/Nrf2 signaling pathway, in a tauopathy mouse model [23]. This might account for DMF’s early (3 h) induction of Hmox1 gene expression, also observed for MHC (Figure 5). Accordingly, we wondered if this Nrf2 activation could be sustained in time (for 24 h) thus contributing to the slightly higher levels (~4.8 fold-increase) of HMOX1 protein in N2a-APPwt cells exposed to DMF, compared to MHC (Figure 6). Since GSK3β is inhibited through phosphorylation at Ser9 by protein kinases such as the protein kinase B (AKT), leading to Nrf2 activation [27,28], phosphorylated levels of AKT and GSK3β were determined by Western blotting 24 h after chemical exposure (Figure 7). According to the results, we did not find any significant differences in AKT (Figure 7A) or GSK3β (Figure 7B) phosphorylated levels, in both N2a-wt and N2a-APPwt cells, at the time point tested (24 h). However, it appears that AKT activation is more robustly sustained by DMF than MHC (Figure 7A). Hence, we should not discard the hypothesis that, in our AD cell model, DMF might inactivate GSK3β more efficiently than MHC, and this could be more evident in a different time point. 

### 2.4. Skin Allergen Modulation of Mitochondrial Membrane Potential and Calcium Basal Levels in N2a Neuronal Cells—Positive Effect of PA and MHC

**Figure 8:**
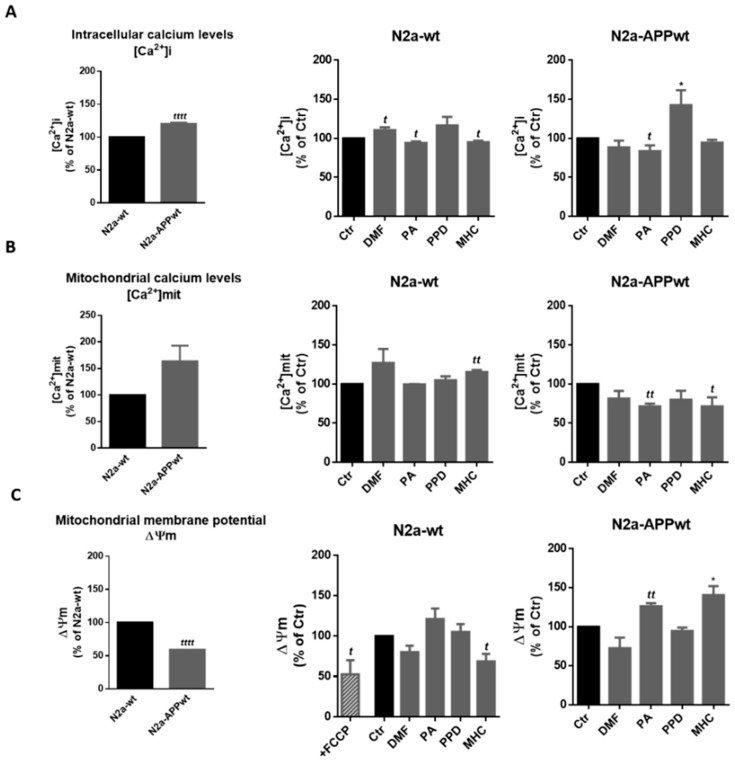
Effect of skin allergens on calcium levels and mitochondrial membrane potential in neuronal cells. Analysis of intracellular ([Ca^2+^]i) (**A**) and mitochondrial ([Ca^2+^]mit) (**B**) calcium levels, and mitochondrial membrane potential (Δψm) (**C**) of N2a (wt and APP-overexpressing) neuronal cells 24 h after chemical exposure. The cells were further incubated with Indo-1, AM for 45 min (**A**), Rhod-2, AM for 45 min (**B**) or TMRE (tetramethylrhodamine ethyl ester) for 30 min (**C**), at 37 °C, protected from light. Values are the mean ± SEM of three independent experiments expressed as the percentage of control (Ctr) cells. Statistics: One-way ANOVA with Dunnett’s multiple comparisons test (*) and unpaired *t*-test (*t*). *p* < 0.05 was considered significant: *^, *t*^ *p* < 0.05 and *^tt^ p* < 0.01, compared to Ctr. Basal levels of [Ca^2+^]i (**A**), [Ca^2+^]mit (**B**) and Δψm (**A**) in untreated N2a neuronal cell lines are depicted in the graphs on the left and expressed as the percentage of N2a-wt cells. Statistics: Unpaired *t*-test. *p* < 0.05 was considered significant: *^tttt^ p* < 0.0001, compared to N2a-wt cells. Legend: DMF—Dimethyl fumarate, PA—Phthalic anhydride, PPD—1,4-Phenylenediamine, MHC—Methyl heptine carbonate, FCCP—carbonyl cyanide 4-(trifluoromethoxy) phenylhydrazone.

Since calcium dyshomeostasis and mitochondrial membrane potential (Δψm) disruption are important hallmarks of AD, we next examined how the skin sensitizers could modulate these events in N2a-wt and APP-overexpressing cells, after 24 h (Figure 8). The cells were loaded with Indo-1/AM, Rhod-2/AM or TMRE (tetramethylrhodamine ethyl ester) probes to evaluate intracellular calcium levels ([Ca^2+^]i), mitochondrial calcium levels ([Ca^2+^]mit) and Δψm, respectively. In the latter experiment, N2a-wt cells were also incubated with FCCP (carbonyl cyanide 4-(trifluoromethoxy) phenylhydrazone), which disrupts mitochondrial membrane potential, to serve as a positive control (for detailed explanation see Section 4). 

Regarding [Ca^2+^]i, our results suggest that N2a-APP overexpressing cells have higher cytosolic Ca^2+^ levels than N2a-wt cells (Figure 8A, left graph), and that these are reduced by PA in N2a-APPwt cells. PA and MHC induced a reduction in [Ca^2+^]mit in N2a-APPwt cells (Figure 8B, N2a-APPwt), which is augmented in N2a-APPwt compared to N2a-wt cells (Figure 8B, left graph). This reduction was accompanied by a recovery of Δψm (Figure 8C, N2a-APPwt) which was significantly impaired in APP-overexpressing N2a cells compared to N2a-wt (Figure 8C, left graph). 

In contrast, DMF did not show any positive effect in reverting the altered calcium levels (mitochondrial and intracellular) or Δψm in the N2a AD cellular model. 

### 2.5. Anti-Inflammatory Profile Evoked by Skin Allergens in BV-2 Microglia Cells

To analyze the anti-inflammatory potential of skin allergens, BV-2 cells were exposed to LPS, a Toll-like receptor 4 agonist and a potent inducer of inflammation. Pro-inflammatory gene and protein expression were subsequently evaluated.

#### 2.5.1. iNos mRNA Levels Decrease Induced by DMF and MHC in LPS-Exposed Microglia

**Figure 9:**
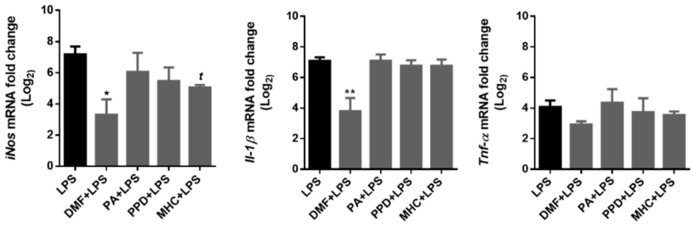
Effect of skin allergens on microglia pro-inflammatory gene expression. BV-2 microglia cells were treated with the chemicals 30 min before treatment with LPS (lipopolysaccharide; 50 ng/mL) for 18 h and iNos, Il-1β and Tnf-α gene expression were evaluated by RT-PCR. Values are the mean ± SEM of three independent experiments expressed relatively to control cells (log_2_ = 0). Statistics: One-way ANOVA with Dunnett’s multiple comparisons test (*) and unpaired *t*-test (*t*). *p* < 0.05 was considered significant: *^, *t*^ *p* < 0.05 and ** *p* < 0.01, compared to LPS. Legend: DMF—Dimethyl fumarate, PA—Phthalic anhydride, PPD—1,4-Phenylenediamine, MHC—Methyl heptine carbonate.

To test the effect of skin allergens on LPS-induced pro-inflammatory gene expression, we exposed BV-2 cells to LPS (50 ng/mL), 30 min after chemical addition. Analysis of the pro-inflammatory genes iNos, Il-1β and Tnf-α were performed 18 h later by real-time RT-PCR (Figure 9). 

DMF strongly decreased the expression of iNos and Il-1β and induced a slight, non-significant decrease in Tnf-α (*p* = 0.07) mRNA evoked by LPS. Moreover, MHC also induced a significant reduction in iNos gene expression, evidencing its anti-inflammatory potential.

#### 2.5.2. Electrophilic Compounds Reduced iNOS Protein Levels in LPS-Exposed Microglia

**Figure 10:**
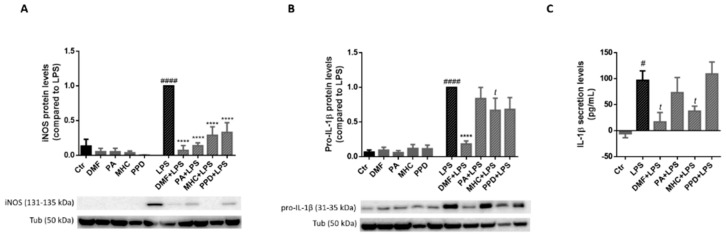
Effect of skin allergens on pro-inflammatory proteins in LPS-exposed microglia. BV-2 microglia cells were treated with the chemicals 30 min before treatment with LPS (50 ng/mL) for 24 h and iNOS (**A**) and pro-IL-1β (**B**) protein levels were determined by Western blotting. Representative images of the blots are shown. Values are the mean ± SEM of four independent experiments normalized for tubulin (Tub) and expressed relatively to LPS. Secreted levels of mature IL-1β were determined in cell supernatants (**C**). Values are the mean ± SEM of three to four independent experiments and are expressed in pg/mL. Statistics: One-way ANOVA with Tukey’s multiple comparisons test (* and #) and unpaired *t*-test (*t*). *p* < 0.05 was considered significant: ^#^ *p* < 0.05 and ^####^ *p* < 0.0001, compared to Ctr; *^t^ p* < 0.05 and **** *p* < 0.0001, compared to LPS. Legend: DMF—Dimethyl fumarate, PA—Phthalic anhydride, PPD—1,4-Phenylenediamine, MHC—Methyl heptine carbonate.

Since mRNA levels of iNos and Il-1β were modulated by the allergens (Figure 9) we next evaluated iNOS and IL-1β protein levels in LPS-exposed BV-2 cells. Thus, the cells were exposed to LPS (50 ng/mL) 30 min after chemicals treatment for 24 h and protein levels were determined by Western blot analysis (Figure 10A,B). As depicted in Figure 10A, all tested chemicals strongly decreased the protein levels of iNOS in LPS-treated cells, suggesting an anti-inflammatory role for these electrophilic molecules. In addition, DMF and MHC were also able to reverse the increase on pro-IL-1β protein levels evoked by LPS (Figure 10B). Hence, we next determined if the mature form of IL-1β was also affected by the allergens, by analyzing IL-1β levels in the cell supernatants, after 24 h of chemical treatment. According to the results, both DMF and MHC induced a significant decrease in IL-1β secreted levels, in LPS-exposed microglia (Figure 10C), thus corroborating our results regarding pro-IL-1β protein levels (Figure 10B). 

## 3. Discussion

AD is the most common cause of dementia mainly affecting humans aged over 60. A disease modifying treatment is desperately needed for AD since the only five drugs recommended for this pathology are completely unable to avoid disease progression. Based on (1) the well-established effect of skin allergens on the activation of Nrf2 [8,18], (2) experimental evidence highlighting Nrf2 as an attractive molecular target in the context of AD [5,6] and (3) the recent approval of the skin allergen DMF for the management of relapsing forms of multiple sclerosis [12], we were led to the innovative hypothesis that DMF and other unexplored skin allergens could have a preventive and/or therapeutic role in AD. 

Therefore, the main goal of this work was to evaluate the effect of safe concentrations (that did not induce cell metabolism impairment and drastic cell death) of skin allergens on three main AD hallmarks: neuronal impaired redox balance, calcium dyshomeostasis and neuroinflammation. To achieve this goal, we used the mouse N2a cell line overexpressing wild-type human APP (N2a-APPwt) as an AD neuronal model, versus wild-type cells (N2a-wt), and the microglia mouse cell line BV-2 exposed to LPS as an in vitro model of AD-associated inflammation.

We selected skin allergens well known for their capacity to react towards cysteine residues and concomitantly to activate the transcription factor Nrf2 [8]. This physical library of allergens, already available in our group, was screened in terms of hydrophobicity (Log P and total surface area), drug-likeness properties (based on the Lipinski rule of five) and toxicity prediction (search for groups classified as PAINS) in order to select a set of compounds able to be absorbed at the gastrointestinal tract, to cross the BBB and to exert low to moderate toxicity. Many of these skin allergens are Michael acceptors suggesting that the presence of an α-β unsaturated carbonyl group is essential for the induction of the Nrf2 pathway [29,30]. Hence, four drug-like electrophilic compounds were used. Besides DMF (as a positive control), we selected two drug-like electrophiles unexplored in the context of AD, PPD and MHC that, similarly to DMF, are Michael acceptors and strongly react with cysteine residues. PA, which mainly reacts with lysine residues, was used as a negative control.

As extensively reviewed elsewhere [24,25], in stressed neural tissues, Hmox1 gene induction is mostly dependent on Nrf2 activation. HMOX1 exerts antioxidant and anti-inflammatory effects, and also induces neurotrophic factor generation. Moreover, HMOX1 deregulation has been associated with the development of neurodegenerative diseases, including AD [24,25]. Hence, we first evaluated the effect of the molecules on Nrf2-dependent gene transcription of Hmox1 and HMOX1 protein levels (Figure 5 and Figure 6, respectively). According to our results, N2a-wt cells expressed higher levels of Hmox1 than N2a-APP overexpressing cells, which corroborates data from the literature reporting that the ROS-dependent mechanism of activating the Nrf2/ARE pathway has become unresponsive in AD models [31] and that AD patients have a decrease in nuclear Nrf2 levels [4]. Of relevance, our results show that PPD and MHC, but not the lysine-reactive PA, were able to increase Hmox1 gene expression in N2a-APPwt cells, which seems to be in accordance with the results regarding allergen-induced Nrf2 activity (Figure 3). However, contradictory data have been reported regarding the beneficial versus harmful effect of HMOX1 upregulation on the neurodegeneration process. Nevertheless, it has also been postulated that the HMOX1 neuroprotective effect is mainly due to the Nrf2-dependent activation, and that its neurotoxic effect may be related to Nrf2-independent activation [24]. This might explain the Nrf2-decreased activity and HMOX1 protein overexpression in the brain of AD patients. Furthermore, this corroborates the data regarding the protective role of both Nrf2 and HMOX1 induction, demonstrated in several cell and animal models of neurodegenerative diseases [24,25]. Accordingly, these data, together with our results concerning Nrf2-induced HMOX1 expression by PPD and MHC, might support the beneficial effect of these allergens in counteracting neurodegeneration in AD. 

Intracellular calcium signaling is of utmost importance in maintaining cellular homeostasis, hence alterations in Ca^2+^ handling have been reported to have a key role in neurodegenerative and age-related diseases, leading to the “calcium hypothesis of aging and AD” [32,33]. Accordingly, several molecular mechanisms have been shown to contribute to amyloid-mediated abnormal Ca^2+^ regulation [33,34,35,36,37,38]. In contrast, others have demonstrated that Ca^2+^ dysregulation might be upstream of the amyloidogenic disease leading to AD development [39,40]. Nevertheless, there are several studies reporting elevated [Ca^2+^]i in AD neurons which, along with alterations in ER/mitochondria contact sites [41,42], might cause [Ca^2+^]mit overload, leading to excessive ROS production and mitochondrial dysfunction. In our study, we also observed increased [Ca^2+^]i (Figure 8A) and reduced Δψm (Figure 8C) in neuronal APP-overexpressing cells (compared to their normal counter-part). Of notice, [Ca^2+^]i was reduced by the lysine-reactive allergen PA in N2a-APPwt cells. Although basal mitochondrial calcium levels between N2a cell lines were not significantly different, a clear tendency to be augmented in N2a-APP compared to wt cells was observed. The sensitizers PA and MHC were able to reduce [Ca^2+^]mit in N2a-APPwt (Figure 8B), which was accompanied by the recovery of the Δψm. Taken together, these results suggest the potential beneficial effect of PA and MHC at restoring Ca^2+^ homeostasis and mitochondrial function in AD. Recently it was demonstrated that the loss of [Ca^2+^]mit efflux accelerates AD development [43]. The authors further suggest that promoting the clearance of pathogenic [Ca^2+^]mit may reduce AD progression, which reinforces the importance of our results. 

Our study also demonstrates the putative anti-inflammatory activity of the chemicals tested, since all of them decreased iNOS expression in LPS-stimulated microglia (Figure 10A). These are relevant results since in a chronic inflammatory response, as in an AD context, there is a sustained high level of iNOS, which seems to play a relevant role in the development of neurodegeneration and/or AD [44,45]. Recently, Zhao and colleagues (2019) demonstrated that sleep disturbances (that lately has been related to an increased risk of AD), lead to memory decline and Aβ42 deposition [46]. Of notice, the Aβ42 deposition was then associated with an upregulation of brain inflammatory mediators, including iNOS, in both the hippocampus and the prefrontal cortex of rats with chronic sleep restriction [47]. 

DMF also reduced iNos mRNA (Figure 9) and Il-1β gene and protein levels (Figure 9 and Figure 10, respectively), which is in accordance with evidence described elsewhere [48], where similar results were observed in primary microglia cells exposed to LPS. Moreover, DMF was also previously reported to rescue neuroinflammation in a Nrf2-independent manner, by decreasing the levels of proinflammatory cytokines in primary microglia derived from Nrf2^−/−^ mice [49]. Regarding the novel electrophilic molecules tested in AD context, our results showed that only MHC was able to induce a decrease in mRNA levels of iNos and in pro-IL-1β protein expression and secretion levels, at least at the time points tested. These results suggest that MHC could have a more robust anti-inflammatory potential compared to PPD. The reduction of proinflammatory cytokines by the molecules tested might be also related to the well-known allergen-induction of IL-4 and IL-13. Although IL-4 and IL-13 are mediators of type 2 associated inflammation, being produced at elevated levels in chronic inflammatory skin diseases [50], both (as well as IL-10) are potent anti-inflammatory cytokines, capable of suppressing proinflammatory cytokine genes such as Il-1 and Tnf [51]. Moreover, IL-4 in particular was shown to regulate brain immunity, with positive effects on neurological disorders [52].

Overall, this work suggests a potential neuroprotective and anti-inflammatory role of the selected skin allergens, which would not normally be considered useful in drug discovery programs, in in vitro models mimicking hallmarks of AD. Although our work was performed in mouse cell models and transformed cells, which can be limiting regarding the interpretation of the results, it supports further studies envisaging the validation of the results using in vivo AD models. Nevertheless, future work should be done regarding the reported results herein, in order to better understand the cellular mechanisms that underlie electrophilic allergen positive effects in AD. In fact, with deeper knowledge, new therapeutic molecules and targets in the context of AD can be designed. Importantly, and as far as we know, our results are the first to suggest a potential capacity of electrophilic allergens in increasing Nrf2-dependent gene expression, restoring calcium homeostasis and mitochondrial function in neurons, and is the first work addressing the effect of skin allergens in microglia cells. 

## 4. Materials and Methods

### 4.1. Materials

The skin sensitizers dimethyl fumarate (DMF), phthalic anhydride (PA), methyl heptine carbonate (MHC) and 1,4-phenylenediamine (PPD), lipopolysaccharide (LPS) from Escherichia coli (serotype O26:B6), Roswell Park Memorial Institute (RPMI)-1640 medium, l-glutamine, MEM non-essential amino acid solution, sodium bicarbonate, d-(+)-glucose, Bovine Serum Albumin (BSA) and the antibody mouse anti-β-Tubulin I were obtained from Sigma-Aldrich (St. Louis, MO, USA). Dulbecco’s Modified Eagle’s Medium (DMEM)-31600 (powder, low glucose with pyruvate; Gibco), fetal bovine serum (FBS; Gibco), geneticin G-418 sulphate (Gibco) and heme oxigenase (HMOX)-1 primary antibody were purchased from Thermo Fisher Scientific (Waltham, MA, USA). Phospho-AKT (Ser473), Phospho-GSK-3β (Ser9), AKT and GSK-3β (D5C5Z) primary antibodies were obtained from Cell Signaling Technologies (Danvers, MA, USA). Aβ40 and Aβ42 Human ELISA Kit (Novex^®^) (Life Technologies), the dyes Rhod-2/AM (Invitrogen), Indo-1/AM (Molecular Probes/Invitrogen) and Hoechst 33342 (Molecular Probes/Invitrogen) were obtained from Alfagene (Lisbon, Portugal). The primary antibodies against inducible nitric oxide synthase (iNOS), interleukin-1 beta (IL-1β), tetramethylrhodamine ethyl ester (TMRE) mitochondrial membrane potential assay kit and Nrf2 Transcription Factor Assay Kit were purchased from Abcam (Cambridge, UK). Dimethyl sulfoxide (DMSO) was purchased from Honeywell (Wabash, IN, USA). Penicillin-streptomycin (100X) was obtained from GRISP Research Solutions (Porto, Portugal). Clarity Western ECL Substrate and horseradish peroxidase Goat Anti-Rabbit IgG (H + L) conjugate were purchased from Bio-Rad (Hercules, CA, USA). The secondary antibody mouse IgG kappa binding protein (m-IgGκ BP) horseradish peroxidase conjugated was obtained from Santa Cruz Biotechnology (Dallas, TX, USA). Protease and phosphatase inhibitor cocktails were from Roche (Mannheim, Germany). Polyvinylidene difluoride (PVDF) membranes were obtained from Millipore Corporation (Bedford, MA, USA). NZYol reagent, iScript Select cDNA Synthesis Kit, NZYSpeedy qPCR Green Master Mix, NZY Colour Protein Marker II were from NZYtech genes and enzymes (Lisbon, Portugal). RNA Storage Solution was from Ambion (Foster City, CA, USA). All primers for reverse transcription polymerase chain reaction were purchased from Eurofins (Nantes, France). Nuclear Extract Kit was obtained from Active Motif (Waterloo, Belgium). The mouse IL-1β/IL-1F2 immunoassay kit was purchased from R&D Systems (Minneapolis, MN, USA). All the other reagents were obtained from Sigma-Aldrich (St. Louis, MO, USA). 

### 4.2. Methods

#### 4.2.1. In Silico Analysis 

The physical library of allergens available in our group was screened in terms of hydrophobicity (Log P and total surface area) [53,54], drug-likeness properties [55] (based on the Lipinski rule of five [56]) and toxicity prediction (search for groups classified as Pan-Assay INterference CompoundS (PAINS) [57]) in order to select a set of compounds that are able to be absorbed at the gastrointestinal tract, to cross the BBB and to exert low to moderate toxicity. 

These parameters were evaluated using Chemdraw software, which provided the SMILE representation of the chemical structures and SwissADME [58], a virtual tool to analyze the physicochemical parameters of hydrophobicity, the hydrogen bond donor and accepting groups and the search for reactive chemical groups that confer high toxicity (the PAINS). 

#### 4.2.2. Cell Culture

BV-2 mouse microglia cell line (ICLC ATL03001, Interlab Cell Line Collection) was cultured and maintained in RPMI-1640 medium supplemented with 10% (*v*/*v*) heat inactivated FBS, 2 mM l-glutamine, 2 g/L sodium bicabornate and 1% (*v*/*v*) penicillin-streptomycin. 

Neuro-2a (N2a) mouse neuroblastoma cell line wild-type (N2a-wt) and the N2a stable overexpressing the human APP wild-type (N2a-APPwt) were a kind gift from Dr. Ciro Isidoro, Università del Piemonte Orientale “A. Avogadro”, Novara, Italy [59]. These cells were cultured in DMEM-31600 medium supplemented with 10% (*v*/*v*) heat-inactivated FBS, 3.7g/L sodium bicarbonate, 4.5 g/L d-glucose, 1% (*v*/*v*) non-essential amino acids and 1% (*v*/*v*) penicillin-streptomycin (N2a-wt) or 0.4 mg/mL geneticin (N2a-APPwt). 

Cells were subcultured every 2–3 days and kept in culture in a 5% CO_2_ incubator at 37 °C for a maximum of 2 months.

#### 4.2.3. Cell Treatment

N2a cells: Cells were plated at a density of 5.3 × 10^4^ cells/cm^2^ and allowed to stabilize overnight (ON). In order to find a non-toxic concentration of chemicals, N2a cells were exposed for 24 h to different concentrations (in mM) of each skin sensitizer (prepared in DMSO) as follows: DMF (0.15, 0.075, 0.05, 0.02 and 0.01), PA (5, 2, 1.75, 1.5, 1 and 0.25), PPD (1.5, 0.5, 0.1, 0.05 and 0.01) and MHC (3, 1, 0.5, 0.1 and 0.01). 

BV-2 cells: Cells were plated at a density of 2.5 × 10^4^ cells/cm^2^ and allowed to stabilize ON. In order to find a non-toxic concentration of chemicals, BV-2 cells were exposed to different concentrations (in mM) of each skin sensitizer (prepared in DMSO) as follows: DMF (1, 0.5, 0.25, 0.2, 0.1, 0.075, 0.05, 0.03, 0.02 and 0.01), PA (5, 3, 1, 0.5 and 0.25), PPD (1, 0.5, 0.25, 0.2, 0.15, 0.1, 0.05, 0.025 and 0.01) and MHC (3; 1.5; 1; 0.5; 0.25 and 0.1), for 24 h.

The final DMSO concentration per well was up to 0.05% (*v*/*v*), which did not affect the cells’ biological parameters evaluated in this study.

Cell metabolism was evaluated through Alamar Blue assay. DMF, PA, PPD and MHC concentrations that did not induce a significant reduction in cell metabolic capacity (compared to the control cells) were determined and further used to assess cell viability through flow cytometry. A non-toxic concentration of the chemicals (that did not reduce cell metabolism and did not induce more than 20% of cell death) was used in the subsequent experiments, as follows:

N2a neuronal cells: DMF (14 µM), PA (500 µM), PPD (10 µM) and MHC (100 µM). The cells were treated with the chemicals for 3 or 6 h (Hmox1 gene expression analysis) and 24 h (HMOX1 protein levels, mitochondrial membrane potential and calcium levels measurement).

BV-2 microglia cells: DMF (30 µM), PA (500 µM), PPD (25 µM) and MHC (200 µM). For the experiments aiming at evaluating the effect of skin sensitizers on BV-2-inflammation model, the chemicals were added to the cells 30 min before the inflammatory stimulus LPS (50 ng/mL). The cells were exposed to the skin sensitizers for 18 h (pro-inflammatory gene expression analysis) or 24 h (protein and IL-1β secretion levels determination). 

#### 4.2.4. Cell Metabolism (Alamar Blue Assay)

To assess cell metabolic activity, the colorimetric Alamar Blue assay was performed, as previously described [60]. Briefly, the cells were plated in duplicates and treated as indicated in the “Cell Treatment” section. Then, 50 µM resazurin solution (in sterile PBS) was added to the cells for 3 h before the terminus of the experiment (i.e., 24 h) and absorbance was read at 570 and 620 nm in a standard spectrophotometer (SLT, Austria). Metabolic active cells are able to reduce resazurin (a non-fluorescent blue dye) into resorufin (pink colored and fluorescent) and, hence, their number correlates with the magnitude of dye reduction. 

#### 4.2.5. Cell Viability (Flow Cytometry)

To further investigate the effect of skin allergens on cell viability, chemical concentrations that did not disturb cell metabolism were further used and cells were analyzed by flow cytometry. BV-2 and N2a treated cells (as previously described in the “Cell Treatment” section) were detached using a cell scraper and collected. After centrifugation at 500× *g* for 5 min to remove medium and cell debris, the supernatant was discarded and the pellet was washed with sterile PBS. After a second centrifugation, the pellet was resuspended in annexin-V buffer (in mM: 140 NaCl, 4 KCl, 0.75 MgCl_2_, 10 HEPES, 2.5 CaCl_2_), pH 7.4. The cells were stained for annexin V (5 ng/mL), a marker for apoptotic cells, and 7-AAD (5 ng/mL), a necrotic cell marker, at room temperature (RT) for 15 min in the dark. Cells incubated at 60 °C for 20 min were used as cell death positive control. The cells (10,000 events) were further analyzed with an BD Accuri C6 cytometer (BD Biosciences, Franklin Lakes, NJ, USA) for viable (annexin-V negative and 7-AAD negative), early apoptotic (annexin-V positive and 7-AAD negative), late apoptotic/necrotic cells population (annexin-V positive and 7-AAD positive) and necrotic cells population (annexin-V negative and 7-AAD positive).

#### 4.2.6. Aβ40 and Aβ42 Peptide Levels Quantification

N2a-wt and N2a-APPwt cells were plated and treated as described in the “Cell Treatment” section. After 24 h the culture medium was replaced with serum-free medium for another 24 h. The supernatant was further collected and centrifuged at 16,000× *g* for 10 min at 4 °C. The levels of Aβ40 and Aβ42 were measured using a sandwich ELISA kit (KHB3481 and KHB3441 from Invitrogen) following the manufacturer’s instructions. The results are expressed in pg/mL.

#### 4.2.7. Nrf2 Activation 

To evaluate if the skin allergens were able to activate the Nrf2 transcription factor in our experimental conditions, N2a-APPwt cells were treated as previously described in the “Cell Treatment” section for 48 h (until reaching ~90% confluence). Cell nuclear extracts were obtained using the Nuclear Extract Kit, according to manufacturer’s instructions. Protein concentration was determined with the bicinchoninic acid method and 5 µg of protein was used to evaluate Nrf2 activation using the Nrf2 Transcription Factor Assay Kit, according to manufacturer’s instructions. The results are expressed as % of untreated control cells (Ctr). The corresponding values of absorbance read in a standard spectrophotometer (SLT, Austria), at an optical density (O.D.) of 450 nm (with a reference wavelength of O.D. 655 nm), are shown in Supplementary Materials (Figure S4). 

#### 4.2.8. Analysis of Gene Expression by Real-Time RT-PCR

Total RNA was extracted from BV-2- and N2a-treated cells (as previously described in the “Cell Treatment” section) with NZYol reagent, according to the manufacturer’s instructions and its concentration was determined by OD260 measurement using a NanoDrop spectrophotometer (Thermo Scientific, Wilmington, DE, USA). Samples were stored in RNA Storage Solution at −80 °C until use. Total RNA (1 µg) was reverse-transcribed using the iScript Select cDNA Synthesis Kit and real-time reverse transcriptase-polymerase chain reaction (RT-PCR) reactions were performed, in duplicate for each sample, on a Bio-Rad MyCycler iQ5 (Hercules, CA, USA), as previously described by us [61]. After amplification, a threshold was set for each gene and Ct values were calculated for all samples. Gene expression changes were analyzed using Bio-Rad CFX Maestro 1.1 system software (Hercules, CA, USA). The results were normalized using Hprt-1 as reference gene. Primer sequences were designed using Beacon Designer software version 7.7 (Premier Biosoft International, Palo Alto, CA, USA) (Table 2) and thoroughly tested.

Since RT-PCR results are presented as ratios of chemical-treated samples over untreated (control) cells, a two-base logarithmic transformation was used to make observations symmetric and closer to a normal distribution. If x represents the fold change of a gene in one sample, then the two-base logarithmic transformation (log_2_(x)) is ln(x)/ln(2). Therefore, fold changes of 2 and 0.5 correspond to mean log_2_ values of 1 and −1, respectively.

#### 4.2.9. Cell Lysates and Western Blotting 

Cells treated as previously described in the “Cell Treatment” section were further collected, centrifuged at 500× *g* for 5 min at 4 °C and washed in ice-cold PBS, pH 7.4. After a second centrifugation, the pellet was incubated in 100 µL of RIPA lysis buffer (50 mM Tris–HCl (pH 8.0), 1% (*v*/*v*) Nonidet P-40, 150 mM NaCl, 0.5% (*w*/*v*) sodium deoxycholate, 0.1% (*w*/*v*) SDS, 2 mM EDTA), freshly supplemented with 1 mM dithiothreitol, and protease (1:7) and phosphatase (1:10) inhibitor cocktails for 30 min in ice. The nuclei and the insoluble cell debris were removed by centrifugation at 4 °C, at 12,000 *g* for 10 min. The obtained extracts, which correspond to supernatant fraction, were collected and stored at −80 °C until use.

Protein concentration was determined using the bicinchoninic acid method and the cell lysates were denatured in 4× concentrated loading buffer (0.25 M Tris pH 6.8, 4% (*w*/*v*) SDS, 200 mM DTT, 20% (*v*/*v*) glycerol and bromophenol blue) at 95 °C, for 5 min.

Briefly, 30 µg of protein was electrophoretically separated on a 10% (*v*/*v*) sodium dodecyl sulphate-polyacrylamide gels (SDS-PAGE) at 130 V for 60–75 min and transferred to a PVDF membrane. The membranes were blocked with 5% (*w*/*v*) non-fat dry milk in Tris-buffered saline ((TBS): 150 mM NaCl, 25 mM Tris-HCl pH 7.6) containing 0.1% (*v*/*v*) Tween 20 (TBS-T) for 1 h, at RT. Membranes were then incubated ON at 4 °C with the primary antibodies against: HMOX1 (1:500), p-AKT (Ser473) (1:1000), pGSK3β (Ser9) (1:500), iNOS (1:1000) and pro-IL-1β (1:500). Following incubation, the membranes were washed for 30 min with TBS-T and incubated with the respective secondary antibodies (anti-rabbit horseradish peroxidase conjugated secondary antibody (1:10,000) or anti-mouse horseradish peroxidase conjugated secondary antibody (1:10,000)), for 1 h at RT. After washing with TBS-T, the membranes were incubated at RT for 1 h with AKT (1:1000), GSK3β (1:1000) or β-tubulin (1:20,000), as loading control. The blots were visualized by chemiluminescence using ImageQuant LAS 500 (GE Healthcare, Chicago, IL, USA). The generated signals were analyzed using total lab TL120 software. 

#### 4.2.10. Intracellular Calcium Levels Measurement 

Intracellular calcium levels ([Ca^2+^]i) were assessed using INDO-1/AM, a cell-permeant and high affinity, intracellular calcium indicator that is ratiometric and UV-light-excitable. After treatment for 24 h with chemicals, cells were washed in Krebs medium without phosphate (in mM: 132 NaCl, 4 KCl, 1.4 MgCl_2_, 6 Glucose, 10 Hepes-Na, 1 CaCl_2_), pH 7.4. Then, N2a cells were incubated with Indo-1/AM (3 µM) for 45 min, at 37 °C, protected from light, washed and maintained in Krebs medium (post-loading), for 15 min, and Indo-1 fluorescence was read (λexc = 350 nm and λem = 410 nm). The cells were then incubated with Hoechst 33342 (0.5 µg/mL; 5 min in the dark) to normalize fluorescence values. 

#### 4.2.11. Mitochondrial Calcium Levels Measurement

Mitochondrial calcium levels ([Ca^2+^]mit) were determined through Rhod-2/AM, a cell permeable calcium indicator that selectively accumulates within mitochondria. N2a cells were exposed to chemicals for 24 h, washed in PBS/0.2% BSA, pH 7.4, and incubated with Rhod-2/AM (10 µM) for 45 min, at 37 °C in the dark. Then, cells were washed and Rhod-2 fluorescence was read (λexc = 552 nm; λem = 581 nm). Cells were further incubated with Hoechst 33342 (0.5 ug/mL; 5 min in the dark) to normalize fluorescence values.

#### 4.2.12. Mitochondrial Membrane Potential Integrity

Mitochondrial membrane potential (Δψm) was evaluated with the TMRE mitochondrial membrane potential assay kit according to the manufacturer’s instructions. Briefly, N2a cells were exposed to chemicals for 24 h and further loaded with TMRE (1 μM) for 30 min, at 37 °C protected from light. A control of the experiment was used by incubating the cells with 50 μM FCCP (carbonyl cyanide 4-(trifluoromethoxy) phenylhydrazone), a protonophore that collapses the Δψm, 10 min before TMRE loading. Then, N2a cells were washed with PBS/0.2% BSA, pH 7.4 and TMRE fluorescence was read (λexc = 549 nm; λem = 575 nm). The cells were also incubated with Hoechst 33342 (0.5 ug/mL; 5 min in the dark) to normalize fluorescence values.

#### 4.2.13. Il-1β Secretion

BV-2 cells were plated and exposed to LPS (50 ng/mL) and to the skin allergens, as previously described (see Section 4.2.3), for 24 h. After, cell culture medium was collected and centrifuged at 16,000× *g* for 10 min at 4 °C. IL-1β levels in the supernatant were determined using a mouse IL-1β Elisa kit, according to the manufacturer’s instructions. Absorbance values were read at O.D. 450 nm (with wavelength correction set to 570 nm). The results were plotted against a standard curve and expressed in pg/mL.

#### 4.2.14. Statistical Analysis

Statistical analysis was performed using GraphPad Prism version 6.0 for Windows (GraphPad Software, San Diego, CA, USA; www.graphpad.com). For each experimental condition, the results are presented as the mean value ± SEM of at least 3 independent experiments. Comparisons between two groups were made by the two-tailed unpaired Student’s *t*-test and multiple group comparisons by one-way ANOVA analysis, with a Dunnett’s or Tukey’s multiple comparison post-test. A value of *p* < 0.05 was considered significant (* *p* < 0.05, ** *p* < 0.01, *** *p* < 0.001, and **** *p* < 0.0001).

## Figures and Tables

**Table 1:** Skin allergens with potential therapeutic application.

Chemical Name	Mechanistic Domains of Chemical Reaction	Oral Absorption	BBB Crossing
Atranol	–	High	Yes
5-Chloro-2-methyl-4-isothiazolin-3-one/MCI	–	High	Yes
4-Nitrobenzyl bromide	–	High	Yes
1,4-Phenylenediamine/PPD	Michael Acceptor	High	Yes
2,4,6-Trichloro-1,3,5-triazine/cyanuric chloride	–	High	Yes
Tetrachlorosalicylanilide/3,3′,4′,5-Tetrachlorosalicylanilide/Tetrachloro-salicylanilide	Acyl Transfer	High	Yes
4-Ethoxymethylene-2-phenyl-2-oxazolin-5-one/oxazolone	–	High	Yes
Squaric acid dibutyl ester	–	High	Yes
Chloroatranol	–	High	Yes
Dimethyl fumarate/DMF	Michael Acceptor	High	Yes
Diphenylcyclopropenone/DP/DCP/DPCP	Acyl Transfer	High	Yes
Benzoyl peroxide	Acyl Transfer	High	Yes
3-Methyl catechol	–	High	Yes
Glutaraldehyde (act. 50%)	Schiff base reagent	High	Yes
Cinnamic aldehyde	–	High	Yes
Methyl heptine carbonate/Methyl 2-octynoate/MHC	Michael Acceptor	High	Yes
Methyl octine carbonate/Methyl 2-nonynoate	Michael Acceptor	High	Yes
*N*,*N*-dimethyl-4-nitrosoaniline	–	High	Yes
Diethyl maleate	–	High	Yes
6-Methyl-3,5-heptadien-2-one	Michael Acceptor	High	Yes
2-Hexylidene cyclopentanone	Michael Acceptor	High	Yes
1,2-Benzisothiazolin-3-one/Proxel active	SN2-reaction at the S-atom proposed	High	Yes
1,2-Dibromo-2,4-dicyanobutane/MDGN/Methyldibromo glutaronitrile	Michael Acceptor	High	Yes
Thimerosal	–	High	Yes
trans-2-Hexenal	Michael Acceptor	High	Yes
Hexahydrophthalic anhydride/1,2-cyclohexane dicarboxylic anhydride	–	High	Yes
Phthalic anhydride/PA	–	High	Yes
5-methyl-2-phenyl-2,4-dihydro-pyrazol-3-one/Phenylmethylpyrazole/A039	–	High	Yes
2-Methoxy-4-methylphenol/Creosol	Michael Acceptor	High	Yes
2-Aminophenol	Michael Acceptor	High	Yes
Isoeugenol	Michael Acceptor	High	Yes
Cinnamyl Alcohol	Michael Acceptor	High	Yes
Farnesol	Michael Acceptor	High	Yes
1,4-Hydrochinone/Hydroquinone	Michael Acceptor	High	Yes
Abietic acid/colophony	Not know	High	Yes
Citral/3,7-Dimethyl-2,6-octadienal	Schiff base reagent	High	Yes
Eugenol/2-Methoxy-4-(2-propenyl)phenol	Michael Acceptor	High	Yes

**Table 2:** List of used primers.

Gene	Forward Primer	Reverse Primer
Hprt-1	5′ GTTGAAGATATAATTGACACTG 3′	5′ GGCATATCCAACAACAAAC 3′
Hmox1	5′ CCAGTTCTACCAGAGTAA 3′	5′ ACAGAAGTTAGAGACCAA 3′
iNos	5′ GCTGTTAGAGACACTTCTGAG 3′	5′ CACTTTGGTAGGATTTGACTTTG 3′
Il-1β	5′ TCTATACCTGTCCTGTGTAATG 3′	5′ GCTTGTGCTTGTG 3′
Tnf-α	5′ CAAGGGACTAGCCAGGAG 3′	5′ TGCCTCTTCTGCCAGTTC 3′

## Supplementary Materials

The following are available online at https://www.mdpi.com/1422-0067/21/20/7791/s1.

## Abbreviations

AD	Alzheimer disease
AKT	Protein kinase B
ARE	antioxidant response element
DMF	Dimethyl fumarate
EMA	European Medicines Agency
FDA	Food and Drug Administration
GSK3β	Glycogen synthase kinase 3 beta
HMOX1	Heme oxygenase 1
iNOS	Inducible nitric oxide synthase
IL-1β	Interleukin 1β
Keap1	kelch-like erythroid cell-derived protein with cap‘n’collar homology-associated protein 1
MHC	Methyl heptine carbonate
Nrf2	Nuclear factor (erythroid-derived 2)-like 2
PA	Phthalic anhydride
PPD	1,4-phenylenediamine
TNF-α	Tumor necrosis factor-α
