# Supplementary material for: Calcium Modulation, Anti-Oxidant and Anti-Inflammatory Effect of Skin Allergens Targeting the Nrf2 Signaling Pathway in Alzheimer’s Disease Cellular Models
Source: Int J Mol Sci. 2020 Oct 21;21(20):7791. doi: 10.3390/ijms21207791 (PMC7594024; doi:10.3390/ijms21207791)
Supplement: Supplementary file 1 [file ijms-21-07791-s001.pdf]

International Journal of
Molecular Sciences

MDPI

Supplementary Methods

Immunocytochemistry

N2a cells were plated in a μ-Slide 8-well ibiTreat *(from Ibidi)* and treated as previously described (see "Cell treatment" section). Then, the cells were washed with ice-cold PBS pH 7.4 and fixed with 4% (*w*/*v*) paraformaldehyde, at RT for 15 min. Cells were further washed three times with PBS and permeabilized with 0.1% (*v*/*v*) Triton X-100 in TBS at RT for 10 min. The cells were then rinsed three times with PBS for 5 min each and incubated with a blocking solution (10% *w*/*v* bovine serum albumin (BSA) in PBS, pH 7.4) at 37 °C for 30 min. After another washing step, the cells were incubated with a primary antibody against HMOX1 (1:100) in 3% (*w*/*v*) BSA, at 37 °C for 2 h. The cells were further washed three times with PBS (pH 7.4) at RT for 5 min each and incubated at 37 °C, for 1 h, with the secondary antibody *anti-mouse IgG (H+L)*, highly cross-adsorbed, *CF*™ 640R (from Sigma-Aldrich, St. Louis, MO, USA), 1:400 in 3% (*w*/*v*) BSA. Next, cells were washed three times with PBS during 5 min each, and counterstained with 0.2 ng/mL *Hoechst 33342* to allow nucleus visualization. After another washing step, the slides were mounted with Ibidi Mounting Medium *(from Ibidi)*. Specificity was evaluated in negative controls by omitting the first antibody. The images were collected by confocal microscopy using a Zeiss LSM 710 (Carl Zeiss AG).

Supplementary Tables and Figures

Table S1. Compiled allergens data.

Chemical Name	CAS Number	Human Potency Category	LLNA Category	Direct Peptide Reactivity Assay	
			EC3	Cysteine	Lysine
2,4,6-Trichloro-1,3,5-triazine/cyanuric chloride	108-77-0	–	Extreme	–	–
4-Nitrobenzyl bromide	100-11-8	–	Extreme	4	1
7,12-Dimethylbenz[α]anthracene	57-97-6	–	Extreme	0	0
Bandrowski's Base/*N*,*N*-bis(4-aminophenyl)-2,5-diamino-1,4-quinone-diimine	20048-27-5	–	Extreme	3	0
Benzoyl peroxide	94-36-0	–	Extreme	4	3
p-Benzoquinone	106-51-4	–	Extreme	4	4
3-Methyl catechol	488-17-5	–	Extreme strong moderate	3	3
4-(N-Ethyl-N-2-methan-sulphonamido-ethyl)-2-methyl-1,4-phenylenediamine/CD3	25646-71-3	–	Strong	4	0
5-methyl-2-phenyl-2,4-dihydro-pyrazol-3-one/Phenylmethylpyrazole/A039	89-25-8	–	Strong	–	–
Benzyl bromide	100-39-0	–	Strong	4	1
Fluorescein-5-isothiocyanate	3326-32-7	–	Strong	4	2
Hexahydrophthalic anhydride/1,2-cyclohexane dicarboxylic anhydride	85-42-7	–	Strong	1	1
Maleic anhydride	108-31-6	–	Strong	4	2
N,*N*-dimethyl-4-nitrosoaniline	138-89-6	–	Strong	4	0

Phthalic anhydride/PA	85-44-9	–	Strong	0	3
Squaric acid diethyl ester	5231-87-8	–	Strong	0	0
Atranol	526-37-4	1	–	–	–
Chloroatranol	57074-21-2	1	–	–	–
Dimethyl fumarate /DMF	624-49-7	1	–	–	–
Pentadecylcatechol	492-89-7	1	–	–	–
1-Chloro-2,4-dinitrobenzene/Dinitrochlorobenzene/DNCB	97-00-7	1	Extreme	4	0
4-Ethoxymethylene-2-phenyl-2-oxazolin-5-one/oxazolone	15646-46-5	1	Extreme	3	2
5-Chloro-2-methyl-1,2-thiazol-3-one	55965-84-9/96118-96-6	1	Extreme	–	–
5-Chloro-2-methyl-4-isothiazolin-3-one/MCI	26172-55-4	1	Extreme	4	1
Diphenylcyclopropenone/DP/DCP/DPCP	886-38-4	1	Extreme	4	0
Tetrachlorosalicylanilide/3,3',4',5-Tetrachlorosalicylanilide/Tetrachloro-salicylanilide	1154-59-2	1	Extreme	1	0
Methylisothiazolinone/MI/2-Methyl-2H-Isothiazol-3-one	2682-20-4	1	Moderate	–	–
Squaric acid	2892-51-5	1	Moderate	–	–
1,4-Phenylenediamine/PPD	106-50-3	1	Strong	4	1
Squaric acid dibutyl ester	2892-62-8	1	Strong	–	–
2-Hexylidene cyclopentanone	17373-89-6	2	–	–	–
6-Methyl-3,5-heptadien-2-one	1604-28-0	2	–	–	–
Isoeugenol	97-54-1	2	–	3	0
Methyl heptine carbonate/Methyl 2-octynoate/MHC	111-12-6	2	–	–	–
Quaternium-15	4080-31-1	2	–	–	–
Tea Leaf Absolute/Epigallocatechol-3-gallate	84650-60-2	2	–	–	–
Thimerosal	54-64-8	2	–	–	–
Thioglycerol/3-Mercapto-1,2-propanediol	96-27-5	2	–	–	–
1,2-Benzisothiazolin-3-one/Proxel active	2634-33-5	2	Moderate	–	–
2-Methoxy-4-methylphenol/Creosol	93-51-6	2	Moderate	–	–
Cinnamic aldehyde	104-55-2	2	Moderate	–	–
Diethyl maleate	141-05-9	2	Moderate	–	–
Dimethylaminopropylamine/3-Dimethyl-amino-1-propylamine	109-55-7	2	Moderate	–	–
Glyoxal (act. 40%)	107-22-2	2	Moderate	–	–
Methyl octine carbonate/Methyl 2-nonynoate	111-80-8	2	Moderate	–	–
trans-2-Hexenal	6728-26-3	2	Moderate	–	–
1,2-Dibromo-2,4-dicyanobutane/MDGN/Methyldibromo glutaronitrile	35691-65-7	2	Strong	4	1

2,5-Diaminotoluene sulfate/2-methylbenzene-1,4-diamine sulfate/Toluene-2,5-diamine sulfate/PTD	615-50-9	2	Strong	3	1
2-Aminophenol	95-55-6	2	Strong	4	1
2-Nitro-1,4-phenylenediamine/Nitro-4-phenylenediamine/2-Nitro-p-phenylenediamine	5307-14-2	2	Strong	4	0
Glutaraldehyde (act. 50%)	111-30-8	2	Strong	1	3
Lauryl gallate/dodecyl gallate	1166-52-5	2	Strong	4	0
Propyl gallate	121-79-9	2	Strong	2	1
Lyral/Hydroxyisohexyl 3-cyclohexene carboxaldehyde	31906-04-4	2	Weak	–	–
Farnesol	4602-84-0	3	–	–	–
Citral/3,7-Dimethyl-2,6-octadienal	5392-40-5	3	Moderate	–	–
1,4-Hydrochinone/Hydroquinone	123-31-9	3	Strong	3	2
4-(Methylamino)phenol sulfate/Metol	55-55-0	3	Strong	4	2
Abietic acid/colophony	514-10-3	3	Weak	4	1
Cinnamyl Alcohol	104-54-1	3	Weak	–	–
Eugenol/2-Methoxy-4-(2-propenyl)phenol	97-53-0	3	Weak	–	–

Legend: LLNA—Local Lymph Node Assay.

Table S2. Scores to the chemicals based on LLNA data and peptide reactivity.

Score	LLNA Data Class	Peptide Reactivity (%)
0	Very weak	< 15
1	Weak	15–40
2	Moderate	40–65
3	Strong	65–90
4	Extreme	> 90

Legend: LLNA—Local Lymph Node Assay.

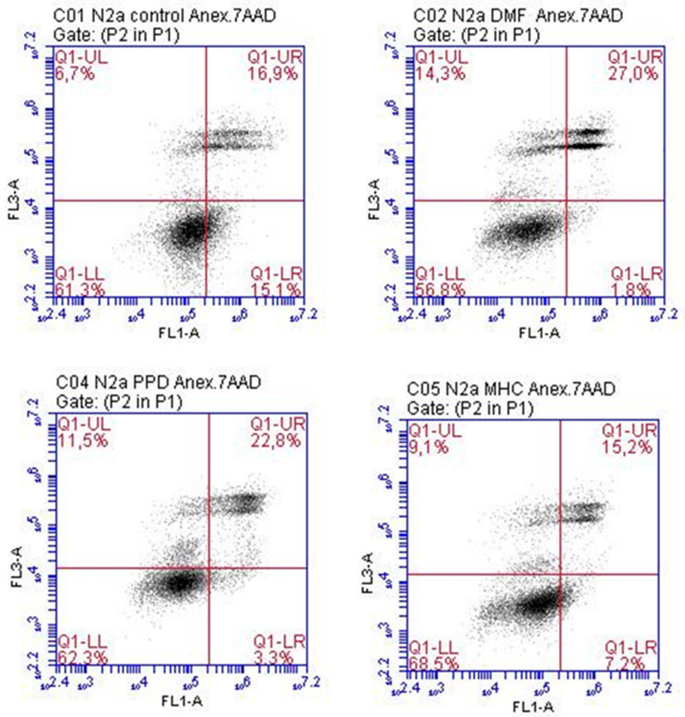

Figure S1. Analysis of N2a-wt cell population viability. Flow cytometry analysis of N2a-wt viable (Q1-LL), early apoptotic (Q1-LR), late apoptotic/necrotic (Q1-UR) and necrotic (Q1-UL) cell populations after exposure to 14 µM of DMF, 500 µM of PA, 10 µM of PPD and 100 µM of MHC for 24 h and stained for Annexin V (apoptotic cells marker) and 7-AAD (necrotic cells marker).

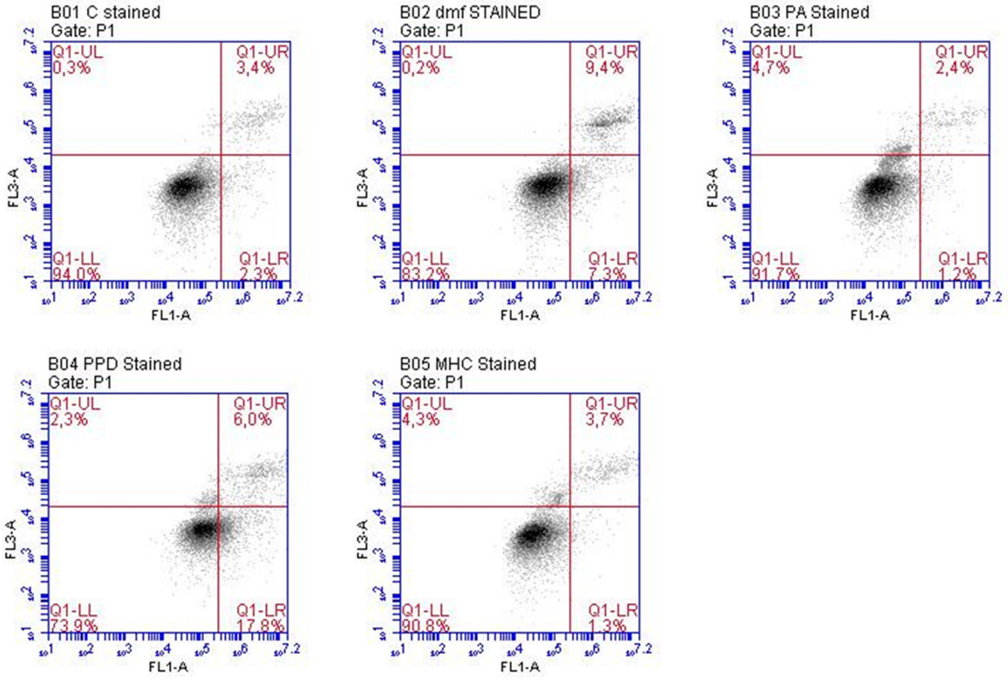

Figure S2. Analysis of BV-2 cell population viability. Flow cytometry analysis of BV-2 viable (Q1-LL), early apoptotic (Q1-LR), late apoptotic/necrotic (Q1-UR) and necrotic (Q1-UL) cell populations after exposure to 30 µM of DMF, 500 µM of PA, 25 µM of PPD and 200 µM of MHC for 24 h and stained for Annexin V (apoptotic cells marker) and 7-AAD (necrotic cells marker).

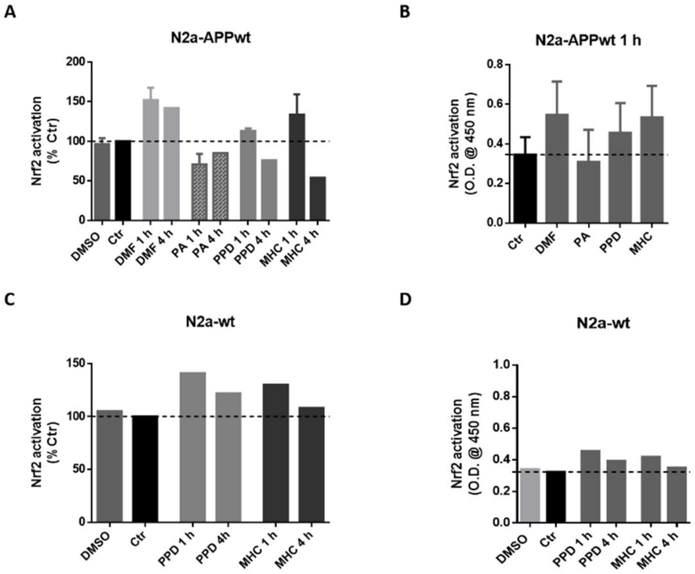

Figure S3. Effect of skin allergens on Nrf2 transcription factor activation in neuronal cells. Nrf2 activation determined in N2a-APPwt (**A** and **B**) and in N2a-wt (**C** and **D**), after chemical exposure, for 1 h and 4 h. Results are expressed as as a percentage of untreated control (Ctr) cells (**A** and **C**). The corresponding absorbance values read at an optical density (O.D.) of 450 nm (with a reference wavelenght of O.D. 655 nm), are depicted in B and D. Values are the mean ± SEM of three independent experiments (**A** and **B**, N2a-APPwt 1 h) or one experiment (**B**–**D**, N2a-APPwt 4 h; N2a-wt 1 h and 4 h). Legend: DMSO—*Dimethyl sulfoxide,* DMF—Dimethyl fumarate, PA—Phthalic anhydride, PPD—1,4-Phenylenediamine, MHC—Methyl heptine carbonate.

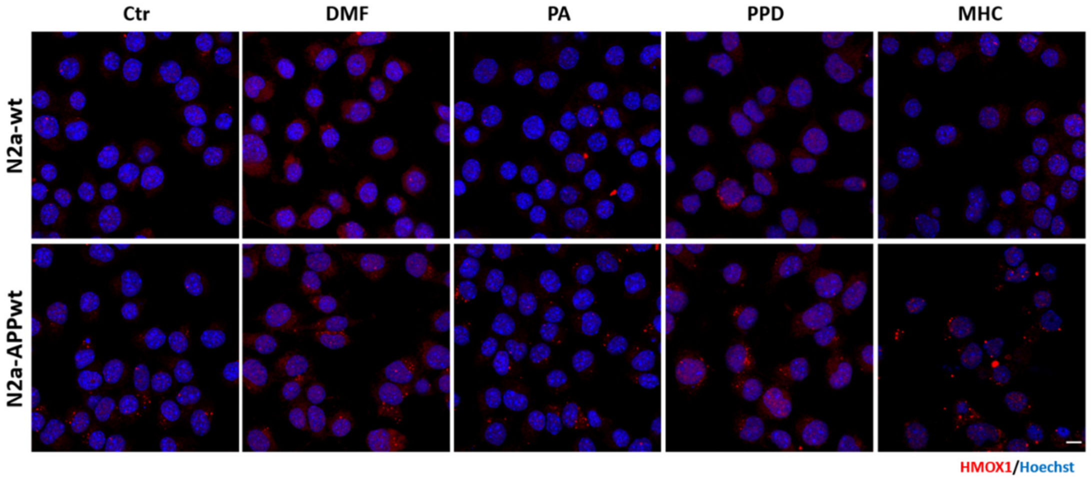

Figure S4. Effect of skin allergens on HMOX1 protein levels in neuronal cells. Representative images of N2a cells immunocytochemistry against HMOX1 (in red), after chemical exposure for 24 h. Nuclei were labelled with Hoechst (in blue). Images were obtained by confocal microscopy with a 60× oil objective. Scale bar = 10 μm (last panel (N2a-APPwt/MHC). Legend: DMF—Dimethyl fumarate, PA—Phthalic anhydride, PPD—1,4-Phenylenediamine, MHC—Methyl heptine carbonate.

© 2020 by the authors. Licensee MDPI, Basel, Switzerland. This article is an open access article distributed under the terms and conditions of the Creative Commons Attribution (CC BY) license (http://creativecommons.org/licenses/by/4.0/).
